# Stage-specific gut microbial restructuring drives estrous transition in rabbits

**DOI:** 10.5713/ab.250529

**Published:** 2025-11-10

**Authors:** Jing Chen, Mingke Gu, Mingrui Zhang, Shihao Wang, Xinyue Zhang, Zhiming Zhu, Qianfu Gan

**Affiliations:** 1 College of Animal Sciences, Fujian Agriculture and Forestry University, Fuzhou, China; 2 Fujian Key Laboratory of Animal Genetics and Breeding, Institute of Animal Husbandry and Veterinary Medicine, Fujian Academy of Agricultural Sciences, Fuzhou, China

**Keywords:** Estrus Regulation, Gut Microbiota, Indole-3-acetic Acid, Rabbit

## Abstract

**Objective:**

This study aimed to investigate the relationship between colonic microbiota and estrous cycle transition in rabbits by integrating 16S rRNA gene sequencing and metabolomic analyses to identify key microbial taxa and metabolites involved in estrus regulation.

**Methods:**

Sixteen female New Zealand white rabbits were divided into diestrus and early estrus groups based on vulvar mucosa color and serum estradiol (E_2_) concentration. Colonic microbiota dynamics were assessed via 16S rRNA sequencing, while metabolomes of colonic contents were profiled using ultra-high performance liquid chromatography–tandem mass spectrometry. Fecal microbiota transplantation (FMT) was performed by oral administration of colonic contents from diestrus or early estrus rabbits to mice with disrupted estrous cycles to evaluate the regulatory effects of microbiota. Exogenous indole-3-acetic acid (IAA) was administered to both mice and rabbits to assess its role in estrus onset and cyclicity restoration.

**Results:**

Colonic microbial composition differed significantly between diestrus and early estrus rabbits. The genera *Anaerostipes* and *Ruminiclostridium* were enriched in early estrus, while the genera *Oscillospirales UCG_010* and *UCG_005* were more abundant in diestrus. FMT from early estrus donors restored cyclicity in mice with disrupted cycles, whereas diestrus FMT did not. Metabolomics identified IAA as a key elevated metabolite in early estrus, and this metabolite accelerated estrus onset and restored cyclicity in both mice and rabbits.

**Conclusion:**

These findings demonstrate that gut microbiota restructuring regulates the estrous transition in rabbits, providing a basis for developing microbiota-targeted strategies to enhance reproductive efficiency in rabbit production and optimize animal reproductive management.

## INTRODUCTION

As typical induced ovulators, female rabbits exhibit a cyclic pattern of mating receptivity over 16–18 days. A dominant follicle secretes estrogen for 12–14 days to drive estrus, which can be divided into three stages: early estrus (slightly swollen, pink vulva), mid-estrus (red, swollen, moist vulva), and late estrus (regressing vulva with reduced swelling and moisture). If ovulation fails, degenerated follicles lead to a 4-day diestrus, characterized by pale white vulvar mucosa [1–3]. The accuracy of estrus detection significantly influences conception success rates. In large-scale livestock production systems, estrus synchronization technology is a critical method for improving reproductive efficiency [4]. Notably, a prolonged photoperiod (16L:8D) has been widely used for estrus synchronization in rabbits [5]. However, the interaction of various endogenous hormones and exogenous factors introduces variability in achieving consistent reproductive outcomes.

The gut microbiome and its metabolites are closely linked to various physiological functions in host animals [6–9], including ovarian function and reproductive capacity [10,11]. A previous study demonstrated significant differences in gut microbial diversity and composition between sows failing to return to estrus post-weaning and their normal cycling counterparts, with these differences correlating with diminished microbial steroid hormone synthesis capacity [12]. Fecal microbiota transplantation (FMT) from healthy rats restored reproductive capacity in two-thirds of polycystic ovary syndrome (PCOS) recipients [13]. Conversely, transplantation of *Aspergillus tubingensis*, a fungus significantly enriched in the gut microbiota of PCOS patients, induced PCOS phenotypes in healthy mice through AT-C1 metabolite-mediated suppression of interleukin-22 secretion by intestinal lymphocytes [14]. These findings collectively reveal that the gut microbiota modulates ovarian function through integrated metabolic, immune, and endocrine pathways. However, the gut microbial dynamics throughout the estrous cycle in rabbits remain poorly characterized.

Thus, the present study aimed to determine whether the gut microbiome was associated with rabbit estrous cycle transition. We characterized differences in the microbiota in colonic contents between diestrus and early estrus rabbits via 16S rRNA sequencing. Subsequently, FMT was performed using colonic contents from rabbits in the diestrus and early estrus groups, respectively, to examine the regulatory role of gut microbiota in estrous cyclicity. Furthermore, untargeted metabolomics was conducted to investigate metabolic differences between diestrus and early estrus rabbits, with the ultimate aim of identifying a key metabolite modulating estrus initiation in female rabbits.

## MATERIALS AND METHODS

### Animals

Female New Zealand White rabbits (4–5 months old, purchased from Anburui Biotechnology) were individually housed in cages under controlled temperature (22±2°C) with *ad libitum* food and water. A subset of rabbits was subjected to long photoperiod (06:00–22:00 daily) to induce estrus, while another subset was maintained under a short photoperiod (06:00–14:00 daily) to sustain diestrus. The first day of stimulation was defined as Day 1 (D1). Female KM mice (6–8 weeks old, purchased from Zolgene Biotechnology) were maintained under the specific pathogen-free conditions at 4–5 mice per cage. Mice were kept on a 14-h light/10-h dark cycle (lights on at 05:00) and fed sterilized food with autoclaved water *ad libitum*.

### Establishment of suppressed estrus mice model

Group-housing female mice leads to the suppression of estrous cycles, a phenomenon known as the Lee-Boot effect [15]. Using this effect, we established a model of estrous cycle inhibition in female mice. Mice were group-housed for at least 10 days before the start of observations. Ten days later, vaginal smears were taken daily for 8 days to select mice with suppressed estrous cycles for subsequent experiments.

### Estrus identification

#### Rabbits

The estrous stage of the female rabbits was determined according to both the vulvar mucosa color and reproductive hormone levels in serum on D1, D3, D5, and D7. Vulva in diestrus is pale and dry, with a tightly closed ostium; vulva in early estrus is slightly swollen and pink, with a slightly opened ostium; vulva in mid-estrus is red, swollen and moist, with a widely opened ostium; vulva in late estrus is purple-black, with decreased swelling and moisture [2,16]. To reduce variability from individual differences, we selected rabbits with consistent color at each time point for follow-up experiments. We also verified the reliability of estrus identification by reproductive hormone detection (see below for details).

#### Mice

Vaginal smears were collected daily at 09:00 for at least two estrous cycles, starting before the first treatment day and continuing after it (oral gavage or intraperitoneal injection). Estrous stages were identified by microscopic analysis of dominant cell types in smears: proestrus (round nucleated epithelial cells), estrus (cornified squamous epithelial cells), metestrus (mixed epithelial cells and leukocytes), and diestrus (nucleated epithelial cells with predominant leukocytes) [17].

### Collection of colonic contents and blood samples

Samples were collected from diestrus and early estrus rabbits. Prior to the collection of colonic contents, 8 blood samples were collected per group. Approximately 2 mL of auricular vein blood was collected in evacuated tubes, centrifuged at 500×g for 10 minutes at 4°C, and the serum was stored in sterile centrifuge tubes at −20°C.

Contents of the distal colon from humanely euthanized rabbits (6 rabbits per group) were rapidly sampled into pre-sterilized cryovials, avoiding contamination with mucosal tissues. Samples were snap-frozen in liquid nitrogen within 30 seconds and stored at −80°C.

### Reproductive hormone detection

Levels of luteinizing hormone (LH), follicle-stimulating hormone (FSH), and estradiol (E_2_) were measured using species-specific ELISA kits: FSH kit (ml027868; Mlbio), LH kit (ml027918; Mlbio), and E_2_ kit (TS-3197; Thousand Sunrise). Optical density (OD) at 450 nm was read using a microplate reader (Infinite F50; TECAN), with blank wells as zero references, and sample concentrations were calculated from the standard curves.

### Microbial DNA extraction and 16S rRNA sequencing

Colonic contents were collected from diestrus and early estrus rabbits. Genomic DNA was extracted using the E.Z.N.A. Stool DNA Kit (D4015; Omega Bio-Tek). DNA purity/concentration was verified via NanoDrop 2000 (Thermo Fisher Scientific). The V3–V4 region was amplified with primers 338F/ 806R and sequenced on Illumina MiSeq PE300 (Illumina).

Raw data were processed using Pear (ver. 0.9.6) for filtering and assembly [18]. Post-assembly, sequences shorter than 230 bp were discarded via Vsearch (ver. 2.7.1) [19], with chimeric sequences further removed through alignment against the Gold Database. High-quality sequences were clustered into operational taxonomic units (OTUs) at 97% sequence similarity via Vsearch. Taxonomic annotations for each OTU were assigned by aligning OTU representative sequences against the Silva138 database [20].

α-diversity indices and β-diversity distance matrices were computed in QIIME (ver. 1.8.0) [21] based on OTU abundance data, followed by principal coordinate analysis (PCoA) analysis and plotting in R (ver. 3.6.0). Linear discriminant analysis effect size (LEfSe) analysis was conducted using Python (ver. 2.7).

### Metabolomics profiling of colonic contents

Twenty-five mg samples were weighed into EP tubes and extracted with 500 μL of a 2:2:1 (v/v/v) methanol:acetonitrile: water extract solution. Following three cycles of homogenization (35 Hz, 4 min) and ice-bath sonication (5 min), extracts were centrifuged (12,000×g, 15 min, 4°C) and supernatants transferred for LC-MS analysis. The quality control samples were prepared by mixing equal aliquots of all supernatants.

Ultra-high performance liquid chromatography (UHPLC) chromatographic separation was performed using an Acquity UHPLC system (Acquity LC; Waters) installed with an ACQUITY BEH Amide column. The mobile phase A was consisted of 25 mM ammonium acetate and 25 mM ammonium hydroxide in water, and the mobile phase B was consisted of 100% ACN. The flow rate was set at 0.5 mL/min. The injection volume was 2 μL, and the samples were maintained at 4°C in the autosampler.

The Orbitrap Exploris 120 mass spectrometer was used for its ability to acquire MS/MS spectra on information-dependent acquisition (IDA) mode in the control of the acquisition software (Xcalibur; Thermo Fisher Scientific). The ESI source conditions were set as following: sheath gas flow rate as 50 Arb, Aux gas flow rate as 15 Arb, capillary temperature 320°C, full MS resolution as 60000, MS/MS resolution as 15000, collision energy: SNCE 20/30/40, spray voltage as 3.8 kV (positive) or −3.4 kV (negative), respectively.

Raw data were converted to mzXML (ProteoWizard) and processed using an XCMS-based R pipeline for peak detection/alignment, missing value imputation (50% minimum value), internal standard normalization, and feature filtering (excluding RSD>30% in QCs), followed by multivariate analysis including principal component analysis (PCA) to assess data distribution and orthogonal partial least squares discriminant analysis (OPLS-DA; MetaboAnalystR) with permutation testing (n = 200) to evaluate model robustness (R^2^/Q^2^), and identification of significant metabolites by variable importance in the projection (VIP)>1, p<0.05 (Student’s t-test), and fold change (FC)>1.5 or <0.67, with pathway analysis using Kyoto Encyclopedia of Genes and Genomes (KEGG) and MetaboAnalyst databases (http://www.metaboanalyst.ca/).

### Fecal microbiota transplantation

FMT was performed according to previous study with slight modification [22]. Briefly, 200 mg of pooled fecal pellets from diestrus and early estrus rabbits, respectively, were homogenized with sterile silica beads in 1.5 mL of saline at 45 Hz for 1 min and filtered through 70 μm strainers. Twenty-seven mice with suppressed estrous cycles were randomly assigned to three groups. Each group received gavage with filtered stool homogenate from diestrus or early estrus donors, or saline, at a dose of 10 μL per gram of body weight, for 5 consecutive days. The estrous cycles of the mice were recorded for 8 days.

### Indole-3-acetic acid treatment

Indole-3-acetic acid (IAA) was dissolved in phosphate buffered saline (PBS) (10 mg/mL stock) using 1 N NaOH, with pH adjusted to 7.4 using 25% (v/v) HCl.

#### Mice

Fifteen mice per group were randomly assigned to three groups: 1) the control group, with daily oral gavage of PBS; 2) the 1.5 mg/kg IAA group, with daily oral gavage of 1.5 mg/kg IAA (I107160; Aladdin); 3) the 3 mg/kg IAA group, with daily oral gavage of 3 mg/kg IAA. Dosages were determined to be safe for mice according to a previous study [23]. Treatments and vaginal smears continued for 8 days.

#### Rabbits

Rabbits were randomly divided into three groups: 1) control group (n = 21), with daily intramuscular injections of PBS; 2) the 0.6 mg/kg IAA group (n = 19), with daily intramuscular injection of 0.6 mg/kg IAA; 3) the 1.2 mg/kg IAA group (n = 22), with daily intramuscular injection of 1.2 mg/kg IAA. Dosages were scaled from mice’s protocols. Treatments commenced on D1 and continued for two days. The color of vulvar mucosa was observed daily to determine estrous stages.

### Statistical analyses

Statistical analysis was performed using the Statistical Package for the Social Sciences (SPSS; ver. 20.0; IBM). Statistical differences in serum reproductive hormones were determined using the unpaired two-tailed Student’s t-test. Statistical differences in the occurrence frequency of different cycle stages in mice with FMT or IAA treatment were determined using the one-way analysis of variance (ANOVA) followed by the Bonferroni method for multiple comparisons. Statistical differences in rabbits treated with IAA were determined using the Pearson’s Chi-squared test. All data are presented as mean±standard error of the mean. Statistical significance was ascertained based on a p-value threshold with * p<0.05 and ** p<0.01.

## RESULTS

### Reproductive hormone dynamics during the estrous cycle

The estrous status of does was determined by characteristic vulvar mucosal morphology, with parallel quantification of serum reproductive hormones. As detailed in Table 1, circulating concentrations of FSH, LH, and E_2_ demonstrated significant stage-specific increases during early estrus compared to those in diestrus female rabbits (p<0.05).

### Gut microbiota restructuring during estrous cycle

**Figure 1:**
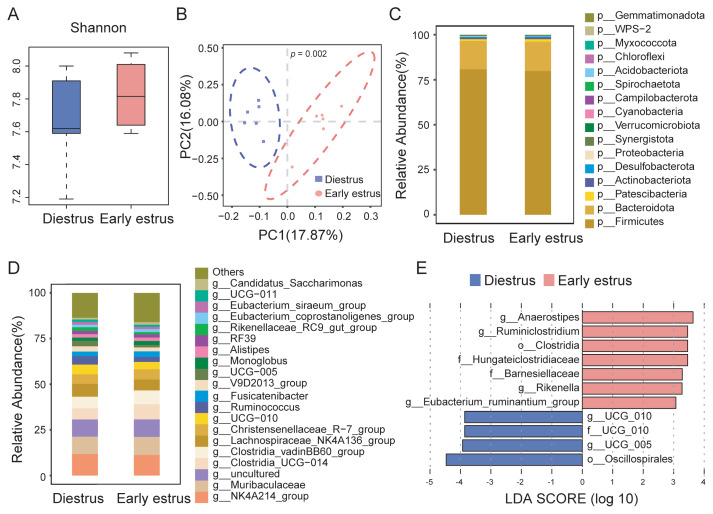
The differences between the colonic flora of early estrus and diestrus female rabbits. (A) α-diversity was performed by Shannon index. (B) β-diversity was performed by principal coordinate analysis (PCoA) based on Bray-Curtis distance. Composition differences of colonic flora between the early estrus and diestrus group at phylum level (C) and genus level (D). (E) Bacterial taxa that differ significantly in abundance in the early estrus and diestrus group, as analyzed by linear discriminant analysis effect size (LEfSe), linear discriminant analysis (LDA)>3, p<0.05.

We next performed 16S rRNA sequencing to characterize the colonic microbiota profiles in rabbits at different estrous stages. α-diversity analysis showed comparable Shannon indices between the early estrus and diestrus phases (p>0.05; Figure 1A). However, Bray-Curtis-based PCoA revealed distinct clustering of microbial communities: diestrus samples were predominantly distributed along the negative PC1 axis (explaining 17.87% variance), while early estrus samples clustered along the positive axis (PERMANOVA: F = 2.06, p = 0.002; Figure 1B).

For microbial composition analysis, we found that at the phylum level, Firmicutes (mean relative abundances: diestrus 80.84%, early estrus 79.88%) and Bacteroidota (mean relative abundances: diestrus 16.30%, early estrus 15.72%) were the dominant phyla in both groups (Figure 1C). At the genus level, *NK4A214_group*, *Muribaculaceae*, *Clostridia_UCG-014*, *Clostridia_vadinBB-60_group*, *Lachnospiraceae_NK4A136_group*, and *Christensenellaceae_R-7_group* (combined mean relative abundances: diestrus 46.07%, early estrus 48.66%) were the dominant bacteria in both groups (Figure 1D).

To further investigate intergroup differences, LEfSe analysis was performed (Figure 1E). At the order level, bacteria of the order Clostridia showed preferential enrichment (linear discriminant analysis>3) in the early estrus does, whereas bacteria of the order Oscillospirales dominated the diestrus microbiota. At lower taxonomic levels, bacteria of six discriminant clades (the families *Hungateiclostridiaceae* and *Barnesiellaceae*, and the genera *Anaerostipes*, *Ruminiclostridium*, *Rikenella*, and *Eubacterium_ruminantium_group*) were enriched in the early estrus group. In contrast, bacteria of the other three discriminant clades: the family *UCG_010* (which belongs to the order Oscillospirales), the genus *UCG_010* (which belongs to family *UCG_010* of the order Oscillospirales), and the genus *UCG_005* (which belongs to the family *Oscillospiraceae* of the order *Oscillospirales*) were enriched in the diestrus group.

### Fecal microbiota transplantation modulates estrous cyclicity

**Figure 2:**
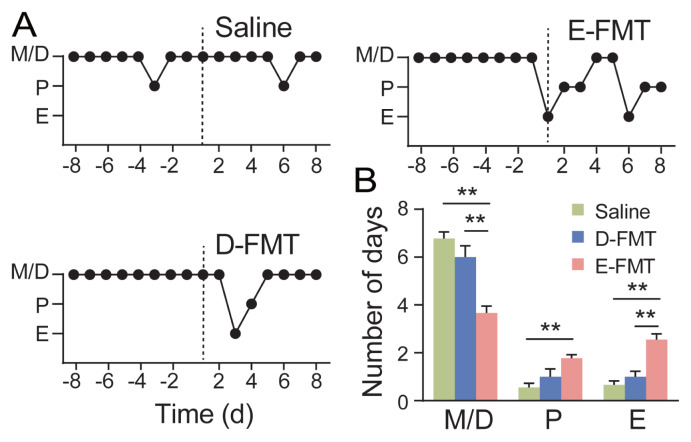
The effect of FMT using rabbit colonic contents on the estrous cycle of mice. (A) Representative estrous cycle of one mouse from each group, the horizontal axis representing days before and after FMT treatment, and the vertical axis representing the stages of the estrous cycle. The dashed lines indicate the initiation time FMT. D-FMT: transplant the colonic contents from diestrus rabbits; E-FMT: transplant the colonic contents from early estrus rabbits. M/D, metestrus/diestrus; P, proestrus; E, estrus. (B) Frequency of occurrence of different cycle stages after FMT in 8 days. Data are presented as mean±SEM. Effects of the treatments were analyzed by the one-way analysis of variance (ANOVA) followed by the Bonferroni posttest. ** p<0.01, n = 9 per group. FMT, fecal microbiota transplantation; SEM, standard error of the mean.

To investigate the impact of gut microbiota on estrous cyclicity, colonic contents from diestrus (D-FMT) or early estrus (E-FMT) donor rabbits were transplanted via oral gavage into female mice with disrupted estrous cycles (see MATERIALS AND METHODS). Recipients receiving D-FMT or the saline control exhibited persistently irregular cycles characterized by prolonged diestrus, as determined by vaginal cytology analysis. In contrast, E-FMT effectively restored regular estrous cyclicity (Figures 2A, 2B). The cycle-restoring capacity of E-FMT—which was absent in D-FMT recipients—indicates that early estrus-phase colonic microbiota contains unique bioactive components sufficient to reinstate ovarian cyclicity.

### Metabolic differences in colonic contents between diestrus and early estrus rabbits

**Figure 3:**
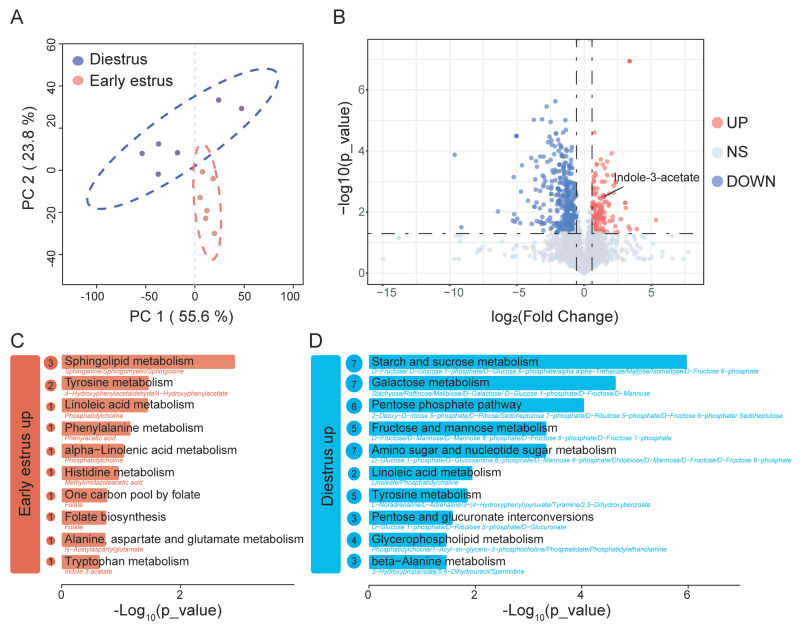
Metabolomic analysis of colonic contents of early estrus and diestrus female rabbits. (A) Display of PCA of the samples in two-dimension (PC1 = 55.6%, PC2 = 23.8%). (B) Volcano plot of differential metabolites between the early estrus and diestrus groups. The blue dots represent the down-regulated differential metabolites, the red dots represent the up-regulated differential metabolites, and the gray dots represent the detected but not significantly different metabolites. VIP>1, p<0.05, FC>1.5 or <0.67. Top 10 up-regulated metabolism and enriched KEGG pathway in the early estrus group (C) and diestrus group (D). PCA, principal component analysis; VIP, variable importance in projection; FC, fold change; KEGG, Kyoto Encyclopedia of Genes and Genomes.

To test the above hypothesis, we performed untargeted metabolomic profiling of colonic contents. The PCA model revealed distinct clustering, indicating a clear separation between the two groups (Figure 3A). Comparative analysis identified 556 differentially abundant metabolites between early estrus and diestrus does, of which 157 were upregulated and 399 were downregulated in early estrus relative to diestrus (Figure 3B, see Supplement 1 for details). Notably, IAA, a tryptophan-derived metabolite primarily generated by gut microbiota, was significantly elevated in the early estrus group (Figure 3B).

Metabolites enriched during early estrus were preferentially enriched in amino acid metabolism pathways (tyrosine metabolism, phenylalanine metabolism, histidine metabolism, alanine, aspartate and glutamate metabolism, and tryptophan metabolism) and lipid metabolism pathways (sphingolipid metabolism, linoleic acid metabolism, alpha-linolenic acid metabolism, glycerophospholipid metabolism, and arachidonic acid metabolism; Figure 3C). Conversely, diestrus-enriched metabolites were significantly associated with carbohydrate metabolic pathways, including starch and sucrose metabolism, galactose metabolism, pentose phosphate pathway, fructose and mannose metabolism, and amino sugar and nucleotide sugar metabolism (Figure 3D).

### Indole-3-acetic acid accelerates estrus onset and restores cyclicity

**Figure 4:**
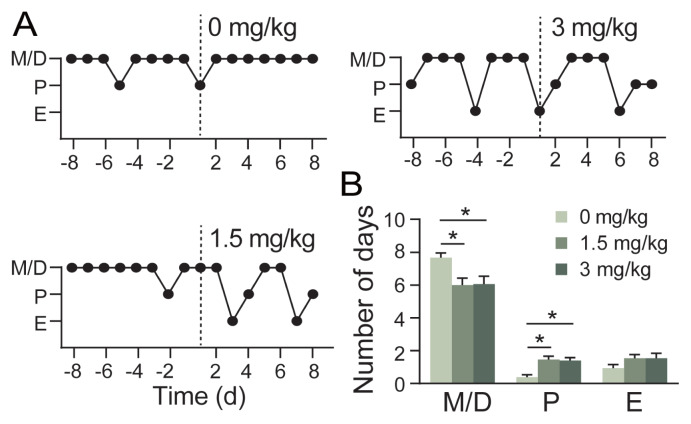
The effects of IAA on the estrous cycle of mice. (A) Representative estrous cycle of one mouse from each group, the horizontal axis representing days before and after IAA treatment, and the vertical axis representing the stages of the estrous cycle. The dashed lines indicate the initiation time of IAA treatment. M/D, metestrus/diestrus; P, proestrus; E, estrus. (B) Frequency of occurrence of different cycle stages after treatments in 8 days. Data are presented as mean±SEM. Effects of the treatments were analyzed by the one-way analysis of variance (ANOVA) followed by the Bonferroni posttest. * p<0.05, n = 15 per group. IAA, indole-3-acetic acid; FMT, fecal microbiota transplantation; SEM, standard error of the mean.

In female mice with disrupted estrous cycles, oral gavage of IAA (1.5 mg/kg or 3 mg/kg) induced rapid transitions to the estrus phase within 3 days (Figure 4A). Vehicle (PBS)-treated controls remained in irregular estrous cycles with a prolonged diestrus phase. After receiving both 1.5 mg/kg and 3 mg/kg IAA, mice exhibited a significantly decreased frequency of the diestrus phase (p<0.05) and a higher frequency of the proestrus phase (p<0.05) compared to the control group (Figure 4B). However, the estrous cycles of 11 out of 15 mice were restored with 1.5 mg/kg IAA treatment, while only 8 of 15 mice achieved restoration of normal estrous cycles following 3 mg/kg IAA treatment.

This pro-estrus transition effect was conserved in rabbits via intramuscular IAA delivery (0.6 mg/kg and 1.2 mg/kg). Does treated with 1.2 mg/kg IAA demonstrated a higher estrus rate than PBS controls and 0.6 mg/kg IAA group at D3 post-injection (Table 2), with concurrent development of vulvar edema.

## DISCUSSION

The gut microbiota and its metabolites have emerged as critical regulators of reproductive performance in livestock. Consistent with prior findings [24,25], the phyla Firmicutes and Bacteroidetes dominated the rabbit intestinal microbiome. Notably, we observed significant alterations in colonic microbiota composition and their associated metabolites between the early estrus and diestrus stages. Among these, the tryptophan-derived metabolite IAA exhibited a significantly higher relative abundance in early estrus compared to that in diestrus.

In early estrus rabbits, the most abundant bacterial taxa included o_Clostridia, *f_ Hungateiclostridiaceae*, *f_Barnesiellaceae*, *g_Anaerostipes*, *g_Ruminiclostridium*, *g_Rikenella*, and *g_Eubacterium_ruminantium_group*. While previous studies reported elevated p_Bacteroidetes, *g_Bacteroides*, and *g_Rikenellaceae_RC9_gut_group* in estrus Hu sheep [26], they also reported estrus-specific p_Bacteroidetes and o_Bacteroidales in buffalo [27]. Such differences likely stem from species-specific variations or sampling site disparities (e.g., rabbit colonic contents *vs*. Hu sheep and buffalo feces). Importantly, all the abovementioned early estrus-enriched genera in rabbits are capable of producing short-chain fatty acids (SCFAs) [28–31] which modulate ovarian function by reducing inflammation and inhibiting granulosa cell apoptosis to prevent follicular atresia [11,32]. However, our study detected no significant differences in SCFA levels between early estrus and diestrus colonic samples, warranting further investigation into their stage-specific roles.

During diestrus, the genera *Oscillospirales UCG_010* and *UCG_005* were significantly enriched. Previous studies have demonstrated that these taxa are positively correlated with fiber degradation. *UCG-010* is not detected in baby elephants fed mainly with milk, while its abundance significantly increased in juvenile and adult elephants that primarily feed on grass and palm [33]. Similarly, compared with Tibetan pigs fed the corn-soybean meal basal diet, those fed the 50% alfalfa-supplemented diet show a significantly increased abundance of *UCG-005*; and the higher fiber utilization capacity of the fecal microbiota from the alfalfa-supplemented group is further verified by *in vitro* fermentation [34]. Consistent with this functional role, our metabolomics revealed that the relative levels of carbohydrate intermediate metabolites, including glucose, D-glucose 1-phosphate and D-fructose 6-phosphate, were significantly higher in diestrus female rabbits compared to those in early estrus individuals. This metabolic shift may be linked to the reduced foraging activity observed during the transition from diestrus to early estrus in female rabbits [2].

IAA, a bioactive indole derivative synthesized via microbial tryptophan metabolism, exerts pleiotropic effects on mammalian physiology, including regulating ovarian function [35–37]. Clinical studies have associated reduced follicular fluid IAA levels with diminished ovarian reserve [38], while *in vitro* evidence shows IAA promotes preantral follicle survival and development [39], underscoring its role in folliculogenesis. Our cross-species (mouse and rabbit) data demonstrate that IAA accelerates estrus transition from diestrus, consistent with these mechanistic insights.

Several bacterial genera have been identified as contributors to the synthesis of IAA from tryptophan, including *Clostridium*, *Lactobacillus*, *Bifidobacterium*, and *Bacteroides fragilis* [40]. Among them, *Clostridium* belongs to the o_Clostridia, and the relative abundance of o_Clostridia was significantly increased in the early estrus group. Although the abundance of the genus *Clostridium* itself showed no significant upregulation in the early estrus group in our results, IAA may instead be synthesized by *Ruminiclostridium*—a genus within the same order (o_Clostridia) that was upregulated. However, no studies have so far confirmed an association between *Ruminiclostridium* and IAA, so additional *in vitro* experiments are required to further verify this hypothesis.

Notably, this study has several limitations. Methodologically, we quantified IAA exclusively in colonic contents, leaving the dynamics of ovarian tissue and serum unexplored across estrous stages. This precludes us from ruling out the possibility that IAA participates in regulatory processes via paracrine or endocrine signaling pathways. To further elucidate IAA’s mechanisms in reproductive regulation, its distribution and fluctuations in ovarian tissue and serum during the estrous cycle should be determined in future work. Moreover, the interspecies consistency of IAA fluctuation patterns requires validation in broader livestock models.

Additionally, hormone measurements included only FSH, LH, and E_2_, excluding progesterone (P_4_). Though evidence in rabbits shows P_4_ has little effect on stimulating estrous behavior [41], it reduces female receptivity during pregnancy [41–44], and this exclusion prevents comprehensive analysis of key reproductive hormones involved in the transition from diestrus to estrus.

## CONCLUSION

In summary, the present study suggests that the composition of colonic microbiota and the profile of colonic metabolites in female rabbits undergo changes concomitant with the estrous cycle, and that the colonic microbiota, together with their metabolites, are involved in the regulation of the estrous cycle.

## Figures and Tables

**Table 1:** Reproductive hormone levels in female rabbits at different estrus stages (n = 8)

Hormones	Diestrus	Early estrus	p-value
FSH (mIU/mL)	44.06±1.62	51.66±0.79	0.0009
LH (mIU/mL)	15.28±0.43	17.23±0.34	0.0032
E_2_ (pg/mL)	322.80±18.53	396.90±25.38	0.0334

Data were represented as mean±SEM.

The data were analyzed by Student’s t-test.

FSH, follicle-stimulating hormone; LH, luteinizing hormone; E_2_, estradiol; SEM, standard error of the mean.

**Table 2:** Number of estrus rabbit with IAA treatment

Times	0 mg/kg		0.6 mg/kg		1.2 mg/kg		χ^2^	p-value

	ES/LE	DI/EE	ES/LE	DI/EE	ES/LE	DI/EE		
D3	4	17	5	14	12	10	6.74	0.034
D5	14	7	18	1	21	1	9.07	0.011
D7	21	0	19	0	22	0	0.00	1.000

The data were analyzed by Pearson’s Chi-squared test.

n = 19–22 per group.

IAA, indole-3-acetic acid; ES, mid-estrus; LE, late estrus; DI, diestrus; EE, early estrus.

## Data Availability

Upon reasonable request, the datasets of this study can be available from the corresponding author.

## SUPPLEMENTARY MATERIAL

**Supplement 1**. Differences in metabolites of colonic contents between early estrus and diestrus rabbits.
