# Supplementary material for: Stage-specific gut microbial restructuring drives estrous transition in rabbits
Source: Anim Biosci. 2025 Nov 10;39(4):250529. doi: 10.5713/ab.250529 (PMC13065002; doi:10.5713/ab.250529)
Supplement: Supplementary file 1 [file ab-250529-Supplementary-1.pdf]

Supplement 1. Differences in metabolites of colonic contents between early estrus and diestrus rabbits

Metabolites	VIP	*p* _value	fold_change	up/down (EE vs. DI)
Lys-Leu	2.12	0.00013	0.0013	down
3-Hexaprenyl-4-hydroxybenzoate	1.40	0.03129	0.0018	down
Undecanoic acid	1.75	0.00948	0.0118	down
Sebacic acid	1.58	0.01890	0.0252	down
2-Aminooctanoic acid	1.99	0.00071	0.0264	down
N-Acetylaspartic acid	1.63	0.01173	0.0271	down
alpha,alpha-Trehalose 6-phosphate	1.62	0.02065	0.0280	down
Phloroglucinaldehyde	2.19	0.00003	0.0304	down
2,3-Dihydroxybenzoic acid (Pyrocatechuic acid)	2.19	0.00003	0.0304	down
2,6-Dihydroxybenzoic acid	2.19	0.00003	0.0304	down
Gentisic acid	2.19	0.00003	0.0304	down
6-Fluoro-1H-indazole-3-carbonitrile	1.67	0.01653	0.0317	down
3-Methylcyclohexanethiol	1.58	0.02272	0.0368	down
Piperonylic acid	1.89	0.00214	0.0384	down
5Z,8Z,14Z-Eicosatrienoic acid	2.08	0.00029	0.0404	down
Stachydrine	1.82	0.00419	0.0551	down
DIHYDROJASMONIC-ACID	2.12	0.00017	0.0655	down
3-Piperidin-4-ylpropanoic acid	1.59	0.02434	0.0671	down
Glucuronic acid	2.23	0.00001	0.0747	down
Mesaconic acid	1.81	0.00663	0.0771	down
Itaconic acid	1.81	0.00663	0.0771	down
3,6,9,12-Tetraoxatetracosan-1-ol	2.02	0.00084	0.0801	down
2,6-Dihydroxybenzaldehyde	1.94	0.00183	0.0824	down
Leucine methyl ester	2.12	0.00021	0.0833	down
7-Hydroxy-1,3-dimethyl-2,4-dioxo-1,2,3,4-tetrahydro-6-pteridinecarbaldehyde	1.80	0.00480	0.0859	down
TRICARBALLYLIC ACID	1.60	0.01966	0.0867	down
Aminoglutethimide	2.07	0.00033	0.0878	down
3-O-Feruloylquinic acid	1.62	0.02076	0.1004	down
Histidinol	1.76	0.00745	0.1019	down
3-[1,1-bis(4-hydroxyphenyl)-3-oxo-3-(2,4,6-trihydroxyphenyl)propan-2-yl]-5,7-dihydroxychromen-4-one	1.88	0.00202	0.1029	down
Flunarizine	2.15	0.00015	0.1031	down
Nipecotic acid	1.60	0.02095	0.1102	down
Ginsenoside Rg6	1.59	0.02420	0.1115	down

Dioscoretine	2.22	0.00001	0.1139	down
M483T256	1.66	0.01424	0.1158	down
Prazepam	2.04	0.00063	0.1180	down
M510T222	1.97	0.00144	0.1217	down
5-Methoxypsoralen	1.48	0.03976	0.1221	down
Kanzonol-F	1.53	0.02376	0.1225	down
Tetradecanedioic acid	2.11	0.00015	0.1229	down
Resorcinol	1.93	0.00124	0.1235	down
D-SEDOHEPTULOSE	1.82	0.00565	0.1237	down
9-O-Methylglyceofuran	1.99	0.00072	0.1255	down
(10E)-6,10-Dimethyl-3-methylidene-2,7-dioxo-2,3,3a,4,5,6,7,11a-octahydro-6,9-epoxycyclodeca[b]furan-4-	1.57	0.02336	0.1286	down
(2,5-Dimethyl-phenylsulfanyl)acetic acid	2.00	0.00097	0.1347	down
Nitrilotriacetic acid	2.27	0.00000	0.1447	down
4,5,6,7-Tetrahydro-1,3-benzothiazole-2,6-diamine	2.12	0.00023	0.1494	down
Methyl 9H-purine-6-carboxylate	1.63	0.01842	0.1530	down
N-Formylaspartate	2.18	0.00005	0.1551	down
4-phenyl-5-(4-pyridyl)-4H-1,2,4-Triazole-3-thiol	1.97	0.00155	0.1559	down
3-Methyl-4-nitro-1-(4-nitrophenyl)-1H-pyrazol-5-ol	2.21	0.00003	0.1676	down
3,5-dimethoxy-alpha-methyl-4-propoxy-Benzeneethanamine	1.59	0.01508	0.1720	down
(+)-Muscarine cation	1.71	0.00883	0.1736	down
Leukotriene-D4	2.10	0.00020	0.1766	down
4-Hydroxy-2,4,6-trimethoxychalcone	1.55	0.02038	0.1773	down
Tuberonic acid	2.23	0.00001	0.1778	down
6-[(1,1-dimethylethyl)sulfonyl]-N-(5-fluoro-1H-indazol-3-yl)-4-Quinolinamine	1.53	0.03529	0.1782	down
Dipiperamide-C	1.87	0.00172	0.1803	down
Fructose	1.88	0.00254	0.1825	down
Tagatose	1.88	0.00254	0.1825	down
[3-(hexadecanoyloxy)-2-[icosa-5.8.11-trienoyloxy]propoxy]({[2.3.4.5.6-	2.20	0.00005	0.1841	down
Melezitose	1.91	0.00146	0.1858	down
M681T273	1.81	0.00403	0.1881	down
Calystegine-C1	2.18	0.00006	0.1891	down
Chitobiose	1.98	0.00098	0.1894	down
(2,5-Dioxotetrahydrofuran-3-yl)acetic acid	1.51	0.03114	0.1936	down
5,7-Dihydroxy-4-phenyl-2H-chromen-2-one	2.04	0.00043	0.1950	down
2,3-Dihydroxy-3-(2-hydroxypropan-2-yl)-8a-methyl-1,2,3a,4,5,8-hexahydroazulene-6-carboxylic acid	2.04	0.00052	0.2067	down

Leu-His	1.55	0.02349	0.2104	down
Thr-Trp	2.15	0.00013	0.2124	down
Cinchonine	2.15	0.00007	0.2204	down
2,2-Dimethylglutaric acid	2.03	0.00041	0.2244	down
2,3-Dinor-11b-PGF2a	1.96	0.00089	0.2248	down
Betaxolol	2.28	0.00000	0.2262	down
1,2-Dilinoleoyl-sn-glycero-3-phosphoethanolamine	1.57	0.02746	0.2314	down
M390T117	1.85	0.00278	0.2331	down
Trehalose	2.21	0.00003	0.2340	down
Maltose	2.21	0.00003	0.2340	down
PE(20:2(11Z,14Z)/14:1(9Z))	1.45	0.04765	0.2402	down
2-Hydroxy-2-methylbutyric acid	1.59	0.02301	0.2410	down
3-Hydroxyvaleric acid	1.59	0.02301	0.2410	down
2-Methyl-3-hydroxybutyric acid	1.59	0.02301	0.2410	down
3-Hydroxyisovaleric acid	1.59	0.02301	0.2410	down
N-[2-(3,4-Dihydroxyphenyl)ethyl]-1-adamantanecarboxamide	1.44	0.04474	0.2423	down
2,3 Dihydroamentoflavone	2.06	0.00047	0.2429	down
1-ethyl-1,3-dihydro-2H-Benzimidazol-2-one	1.44	0.04671	0.2432	down
N-((7E,11Z)-6,13,14-Trihydroxy-3-(1H-indol-3-ylmethyl)-4,5,10,12-tetramethyl-1,17-dioxo-	1.81	0.00554	0.2466	down
M441T103	1.71	0.00839	0.2478	down
5-Fluoro-2-hydroxypyridine	1.36	0.04858	0.2510	down
D-Allose	1.74	0.00943	0.2514	down
M589T264	1.51	0.03071	0.2564	down
Spermidine	1.46	0.04368	0.2572	down
4-Hydroxy-2-methylacetophenone	1.92	0.00168	0.2572	down
4-Allylcatechol	1.92	0.00168	0.2572	down
4-Hydroxy-3-methylacetophenone	1.92	0.00168	0.2572	down
2-Hydroxy-4-methylacetophenone	1.92	0.00168	0.2572	down
2,6-Dimethyl-4-hydroxybenzaldehyde	1.92	0.00168	0.2572	down
2-Hydroxy-5-methylacetophenone	1.92	0.00168	0.2572	down
3-Phenylpropanoic acid	1.92	0.00168	0.2572	down
14-O-Acetyldaunomycinone	1.86	0.00259	0.2573	down
Didecyl hydrogen phosphate	1.86	0.00436	0.2579	down
Pesticide3-Carbofuran-C12H15NO3-Furadan	1.39	0.04223	0.2589	down
Aleuritic acid	2.18	0.00004	0.2609	down

PC(18:1(9Z)/18:3(9Z,12Z,15Z))	2.12	0.00010	0.2669	down
Taxifolin	1.79	0.00506	0.2704	down
2-amino-3-({hydroxy[2-(icosanoyloxy)-3-[octadec-11-enoyloxy]propoxy]phosphoryl}oxy)propanoic acid	1.82	0.00419	0.2726	down
Irinotecan	1.87	0.00189	0.2732	down
D-Ribose	1.84	0.00453	0.2742	down
3,11-Dihydroxy-7-drimen-6-one	1.89	0.00278	0.2743	down
M433T161	1.96	0.00110	0.2798	down
Benzoic acid	2.09	0.00028	0.2800	down
3-hydroxybenzaldehyde	2.09	0.00028	0.2800	down
4-Hydroxybenzaldehyde	2.09	0.00028	0.2800	down
p-Toluquinone	2.09	0.00028	0.2800	down
LysoPC(18:1(11Z))	2.14	0.00006	0.2807	down
3,3,3-Trifluoroalanine	1.58	0.02262	0.2811	down
Xanthine	1.50	0.02643	0.2829	down
Oxypurinol	1.50	0.02643	0.2829	down
Mometasone-furoate	2.05	0.00045	0.2831	down
DG(22:5(4Z,7Z,10Z,13Z,16Z)/20:3(5Z,8Z,11Z)/0:0)	1.47	0.03293	0.2848	down
2-Deoxyuridine	1.94	0.00195	0.2862	down
Resveratrol	1.94	0.00195	0.2862	down
LIMONIN	1.98	0.00076	0.2863	down
Deacylgymnemic acid	1.87	0.00399	0.2876	down
N-[(1S,2R,3E)-2-hydroxy-1-(hydroxymethyl)-3-heptadecen-1-yl]-6-[(7-nitro-2,1,3-benzoxadiazol-4-yl)amino]-	1.51	0.03184	0.2879	down
Nateglinide	1.50	0.02868	0.2897	down
Pyridoxine	1.81	0.00560	0.2917	down
Norepinephrine	1.81	0.00560	0.2917	down
(10E,15Z)-9,12,13-Trihydroxyoctadeca-10,15-dienoic acid	1.48	0.03407	0.2924	down
3-Indoleacetonitrile	2.02	0.00077	0.2929	down
Isoglobotriaose (iGb3)	2.00	0.00107	0.2994	down
3-(Hepta-1,3-dienyl)hexanedioic acid	1.49	0.02495	0.3033	down
10,11-Epidioxycalamene	2.13	0.00007	0.3053	down
apigenin 6,8-digalactoside	2.16	0.00008	0.3069	down
Dodecanedioic acid	2.10	0.00013	0.3096	down
Montecristin	1.87	0.00316	0.3103	down
(12Z)-9,10,11,14,15,16,17,18,19,20,21,22-Dodecahydro-5,8-ethenobenzo[20]annulene-2,4,6,24-tetrol	2.11	0.00015	0.3137	down

2-(1,2-Dihydroxypropan-2-yl)-6a-hydroxy-8,9-dimethoxy-1,2,12,12a-tetrahydrochromeno[3,4-b]furo[2,3-h]chromen-6(6aH)-one	1.80	0.00725	0.3156	down
PC(44:11)	1.54	0.01981	0.3159	down
Saccharin	1.50	0.02493	0.3181	down
4-Ipomeanol	1.85	0.00459	0.3187	down
2-Hydroxy-3-isopropyl-6-methylbenzoic acid	2.13	0.00014	0.3241	down
Lys-Val	1.40	0.04073	0.3242	down
L-Glutamate-5-semialdehyde	1.67	0.01433	0.3261	down
N-Acetylhistamine	1.48	0.03472	0.3280	down
Lactate	1.89	0.00276	0.3363	down
3-Hydroxypropionic acid (beta-lactic acid)	1.89	0.00276	0.3363	down
Maltotetraose	1.53	0.03228	0.3365	down
15-Oxo-11Z,13E-eicosadienoic acid	2.21	0.00002	0.3386	down
4-Nitrophenol	2.04	0.00040	0.3418	down
3-Chloro-4-hydrazinophenylmethylsulfonylmethyl sulfone	1.98	0.00116	0.3430	down
2,4-Pyridinedicarboxylic acid	2.05	0.00034	0.3446	down
(24E)-12,15-Dihydroxy-3-(pentopyranosyloxy)-9,19-cyclolanost-24-en-26-oic acid	1.40	0.04779	0.3448	down
M483T224	2.06	0.00034	0.3451	down
1-Palmitoyllysophosphatidate	2.10	0.00015	0.3454	down
N-Benzoyl-N-(2-pyridinyl)thiourea	1.76	0.00638	0.3486	down
2-Chloro-2-hydroxy-5-methylbenzophenone	1.59	0.02485	0.3500	down
Xanthotoxol	2.02	0.00067	0.3525	down
1-Kestose	2.09	0.00030	0.3532	down
1-Butylimidazole	1.59	0.02119	0.3550	down
Isocarbamid	1.83	0.00390	0.3551	down
gamma-Linolenic acid	1.99	0.00054	0.3572	down
alpha-Linolenic acid	1.99	0.00054	0.3572	down
EDTA	1.70	0.01222	0.3574	down
Maltotriose	2.07	0.00044	0.3585	down
Raffinose	2.02	0.00087	0.3594	down
Val-Leu	1.91	0.00203	0.3597	down
Raltitrexed	1.82	0.00609	0.3618	down
2,4-Dihydroxybutanoic acid	2.11	0.00024	0.3619	down
N-(2,5-Dihydroxyphenyl)acetamide	1.64	0.01605	0.3622	down
M163T255	1.88	0.00311	0.3644	down

cis-5-Dodecenoic acid	1.70	0.01275	0.3668	down
Inosine	1.66	0.01818	0.3707	down
Phe-Met-Arg-Phe-amide	1.43	0.03761	0.3739	down
Stachyose	1.58	0.02589	0.3741	down
Ala-Glu	1.49	0.03636	0.3752	down
Ribulose 5-phosphate	1.95	0.00126	0.3772	down
7-Hydroxy-5-methylflavone	2.22	0.00001	0.3793	down
DOCOSANOL	1.56	0.02522	0.3813	down
5-(3,4-Dihydroxy-4,4,7,8a-tetramethyl-6-oxospiro[2,3,4a,5,6,7-hexahydro-1H-naphthalene-8,2-3,8-dihydrofuro[2,3-e]isoindole]-7-yl)pentanoic acid	1.70	0.00904	0.3871	down
(9Z,12E)-15,16-Dihydroxyoctadeca-9,12-dienoic acid	2.20	0.00002	0.3884	down
3-Methoxy-4-(1,3-oxazol-5-yl)aniline	2.09	0.00024	0.3892	down
M584T53	1.73	0.00824	0.3905	down
1-Ethyl-3-methylimidazolium cation	2.00	0.00077	0.3926	down
Deoxyinosine	2.14	0.00009	0.3932	down
N-(4-Chloro-3-nitrophenyl)-3-methylbenzamide	2.00	0.00103	0.3956	down
2-(4-Methoxyphenyl)quinazolin-4-ol	2.11	0.00015	0.3968	down
3-(4-Hydroxyanilino)-5,5-dimethyl-2-cyclohexen-1-one	1.75	0.00866	0.3983	down
Linoleate	1.91	0.00173	0.3988	down
4-Aminobutane-2-thiol	1.86	0.00344	0.3989	down
Diuron-desmethyl	2.01	0.00094	0.3996	down
3,4-Dihydroxymandelate	1.63	0.01757	0.4013	down
Glucose 1-phosphate	1.94	0.00181	0.4024	down
Fructose 6-phosphate	1.94	0.00181	0.4024	down
2-(Hydroxymethyl)-5-methyl-4H,7H-[1,2,4]triazolo[1,5-a]pyrimidin-7-one	1.85	0.00500	0.4024	down
2-Methoxynaphthalene	1.71	0.01254	0.4027	down
Maltopentaose	1.56	0.02904	0.4031	down
D-myo-Inositol-4-phosphate	1.92	0.00218	0.4046	down
M451T270	1.61	0.01439	0.4062	down
LPA(18:0)	1.69	0.01167	0.4069	down
2-Aminoheptanoic acid	1.81	0.00659	0.4072	down
Astemizole	1.79	0.00699	0.4073	down
Epiafzelechin (2R,3R)(-)	2.15	0.00010	0.4074	down
Val-Ile	1.93	0.00183	0.4075	down
Leu-Val	1.93	0.00183	0.4075	down

3-isopropyl-1,2-benzenediol	1.80	0.00507	0.4092	down
Glucose 6-phosphate	1.97	0.00132	0.4105	down
Mannose 6-phosphate	1.97	0.00132	0.4105	down
Fructose 1-phosphate	1.97	0.00132	0.4105	down
Mannose 1-phosphate	1.97	0.00132	0.4105	down
CPA(18:0)	1.60	0.01753	0.4109	down
M226T188	1.53	0.02455	0.4118	down
Penicillic acid	1.67	0.01387	0.4147	down
5-Phenylisoxazol-3-ol	1.61	0.01396	0.4151	down
N-(4-Chlorobenzyl)-9H-purin-6-amine	1.56	0.02149	0.4157	down
Methylsuccinic acid	1.87	0.00198	0.4179	down
Ethylmalonic acid	1.87	0.00198	0.4179	down
Isomucronulatol	1.78	0.00820	0.4183	down
3,6-Dichloro-N-(pyridin-2-ylmethyl)pyridazine-4-carboxamide	1.85	0.00406	0.4187	down
5-Amino-2,3-bithiophene-4-carboxamide	1.92	0.00222	0.4189	down
2-Phthalimidoglutaric acid	1.90	0.00277	0.4197	down
Neomangiferin	1.72	0.01051	0.4205	down
Sedoheptulose-7-phosphate	1.61	0.01717	0.4248	down
6-(Thiophen-2-yl)imidazo[2,1-b][1,3]thiazole	1.93	0.00202	0.4249	down
4,4,4-Trifluoro-1-(5-methylthiophen-2-yl)butane-1,3-dione	2.00	0.00111	0.4252	down
(S)-Carnitine	1.48	0.03733	0.4267	down
Citronellyl-beta-sophoroside	1.85	0.00308	0.4276	down
Ethiozin	1.62	0.02062	0.4281	down
CPA(18:1(9Z)/0:0)	1.78	0.00663	0.4288	down
1H-Pyrrole-3-carboxylic acid	1.86	0.00245	0.4304	down
L-Valyl-L-phenylalanine	1.77	0.00767	0.4322	down
PC(18:3(6Z,9Z,12Z)/15:0)	1.70	0.00885	0.4337	down
12-Hydroxy-4,4a,6a,7-tetramethyldocosahydrospiro[naphtho[2,1:4,5]indeno[2,1-b]furan-8,2-pyran]-2-yl 6-O-hexopyranosylhexopyranoside	1.94	0.00147	0.4339	down
1-Isonicotinoyl-4-piperidinylamine	2.05	0.00049	0.4358	down
9-Hydroxynonanoic acid	1.89	0.00221	0.4359	down
8-Iodoquinazolin-4-ol	1.42	0.04435	0.4378	down
Ataluren	1.87	0.00334	0.4400	down
[1,2,4]Triazolo[1,5-a]pyrimidine-2-carboxylic acid, 1,7-dihydro-5-methyl-7-oxo-, ethyl ester	1.57	0.02773	0.4403	down
(2E)-6-((3,4-Bis-O-((2E)-6-hydroxy-2,6-dimethylocta-2,7-dienoyl)-beta-D-glucopyranosyl)oxy)-2,6-dimethylocta-2,7-dienoic acid	1.48	0.04137	0.4415	down

2-Hexyl-3-phenyl-2-propenal	1.80	0.00475	0.4432	down
Echinocystic acid 3-glucoside	1.53	0.02899	0.4433	down
Maltol	1.93	0.00210	0.4450	down
CROTONIC ACID	1.77	0.00778	0.4452	down
Meprobamate	1.78	0.00836	0.4455	down
Thymidine	1.72	0.01180	0.4481	down
Lapachol	1.72	0.01180	0.4481	down
3-Pyridinepropionic acid	1.98	0.00135	0.4495	down
M404T224	1.76	0.00580	0.4504	down
3,5-dihydroxydecanoic acid	1.68	0.00809	0.4508	down
Melibiose	1.86	0.00291	0.4530	down
Isomaltose	1.86	0.00291	0.4530	down
M680T119	1.52	0.02676	0.4546	down
Galangin	2.01	0.00055	0.4577	down
6-Azathymine	1.57	0.02841	0.4598	down
Glucose	2.06	0.00031	0.4623	down
Galactose	2.06	0.00031	0.4623	down
Mannose	2.06	0.00031	0.4623	down
Anhydrotetracycline	1.85	0.00464	0.4623	down
M501T251	1.76	0.00728	0.4631	down
M455T114	1.43	0.04640	0.4638	down
Methysticin	1.77	0.00771	0.4646	down
M181T115	1.58	0.02136	0.4665	down
1-Oleoyl-sn-glycerol 3-phosphate	1.90	0.00229	0.4681	down
1-{[3-(Trifluoromethyl)phenyl]sulfonyl}-4-piperidinecarboxylic acid	2.04	0.00038	0.4719	down
Sophoraside A	1.63	0.01966	0.4743	down
Bindone	1.97	0.00100	0.4750	down
4-Chloro-N-(4-methylphenyl)-2-nitrobenzamide	1.95	0.00111	0.4752	down
Glycylleucine	1.67	0.01785	0.4755	down
2-(HYDROXYMETHYL)BUTANOIC ACID	1.86	0.00428	0.4802	down
4-Cholesten-3-one	1.44	0.04101	0.4894	down
5-(2-methoxyethylamino)-3H-1,3,4-thiadiazole-2-thione	1.72	0.00743	0.4910	down
2-[(3,5-difluoro-4-hydroxyphenyl)amino]-7,8-dihydro-5,7-dimethyl-8-(3-methylbutyl)-6(5H)-Pteridinone	1.59	0.02086	0.4917	down
1,2-Dipalmitoleoyl-sn-glycero-3-phosphoethanolamine	1.46	0.03141	0.4919	down
3-hydroxy-6-(4-hydroxyphenoxy)-Spiro[isobenzofuran-1(3H),9-[9H]xanthen]-3-one	1.82	0.00605	0.4929	down

PC(15:0/P-18:0)	1.48	0.02865	0.4941	down
Pheophorbide a	1.67	0.00637	0.4945	down
3-Hydroxydecanoic acid	1.63	0.01556	0.4962	down
Leu-Met	1.46	0.04067	0.4982	down
PE(18:0/18:2)	1.54	0.02479	0.5000	down
2-Deoxyribose 5-phosphate	1.77	0.00558	0.5001	down
Ecgonine-methyl-ester (EME)	2.07	0.00035	0.5006	down
cis-7-Hexadecenoic acid	2.06	0.00026	0.5006	down
18-alpha-Glycyrrhetinic acid	1.62	0.02007	0.5029	down
Euscaphic acid	1.62	0.02007	0.5029	down
Val-Val	1.77	0.00631	0.5038	down
Rhamnose	1.92	0.00192	0.5041	down
Andrographiside	1.47	0.03423	0.5043	down
Azepane-1-carboxylic acid (4-chlorophenyl)amide	1.56	0.01658	0.5049	down
Cytidine	1.53	0.03494	0.5050	down
1,3-Dimethyluracil	1.70	0.01253	0.5053	down
Deoxyadenosine	1.57	0.01733	0.5058	down
5-Deoxyadenosine	1.57	0.01733	0.5058	down
Cordycepin	1.57	0.01733	0.5058	down
N-(4-Piperidinyl)acetamide	1.67	0.01363	0.5061	down
Deoxyguanosine	1.57	0.02386	0.5068	down
LPC(18:2/0:0)	1.43	0.04216	0.5080	down
Glutaric acid	2.14	0.00010	0.5085	down
Coenzyme-Q4	2.10	0.00019	0.5087	down
Acetyl-L-threonine	1.51	0.03675	0.5102	down
3,3,4,4-Tetrahydroxy-5,5-diisopropyl-2,2-dimethylbiphenyl	1.45	0.04290	0.5138	down
Acidissiminin-epoxide	1.79	0.00773	0.5158	down
Creatine, ethyl ester	1.43	0.04345	0.5162	down
Pyridoxal (Vitamin B6)	1.59	0.02051	0.5163	down
Pseudouridine	2.04	0.00055	0.5187	down
3-(2-Hydroxyphenyl)propanoic acid	1.63	0.02237	0.5195	down
4-Ethoxybenzoic acid	1.63	0.02237	0.5195	down
Ethyl 4-hydroxybenzoate	1.63	0.02237	0.5195	down
Ethyl 3-hydroxybenzoate	1.63	0.02237	0.5195	down
3-Methoxyphenylacetic acid	1.63	0.02237	0.5195	down

Glucosamine 6-phosphate	1.83	0.00465	0.5196	down
Leu-Ile	1.82	0.00532	0.5197	down
Chrysin Dimethyl Ether	1.45	0.03968	0.5215	down
Coniferyl aldehyde	1.53	0.02625	0.5238	down
6-(2-Pyridinyl)-1,3,5-triazine-2,4-diamine	1.87	0.00251	0.5241	down
Cumaroylspermidine	1.71	0.01346	0.5246	down
Mycothiol	1.53	0.03126	0.5256	down
(1alpha,2alpha,3beta,5Xi,9Xi,13Xi,14Xi,18Xi)-1,2,3,22,23,29-Hexahydroxy-13,27-cycloolean-11-en-28-oic acid	1.51	0.03202	0.5260	down
N-Ethylglycine	1.64	0.01339	0.5289	down
Quinic acid	1.63	0.02195	0.5290	down
3-Aminobenzoic acid	1.58	0.02151	0.5297	down
Rhamnazin	2.12	0.00007	0.5303	down
M306T135	1.55	0.02261	0.5308	down
cis-2,3-Dimethylthiirane	1.64	0.01603	0.5315	down
Hydroxy(phenyl)2-thienylacetic acid	1.97	0.00109	0.5332	down
4-[4-(1,3-benzodioxol-5-yl)-5-(2-pyridinyl)-1H-imidazol-2-yl]-Benzamide	1.89	0.00399	0.5343	down
N-Acetylcysteinamide	1.55	0.01971	0.5372	down
Tyramine	1.76	0.00536	0.5387	down
N-[[4-(1,4,8,11-tetraazacyclotetradec-1-ylmethyl)phenyl]methyl]-2-Pyridinemethanamine	1.43	0.03784	0.5389	down
4-Hydroxy-N-(4-hydroxyphenyl)benzamide	1.66	0.01507	0.5390	down
Ethyl hydrazinoacetate	1.49	0.03389	0.5408	down
(1,3,5-Trimethyl-1H-pyrazol-4-yl)methanol	2.06	0.00036	0.5434	down
(2R)-3-Hydroxyisovaleroylcarnitine	1.68	0.01264	0.5486	down
5-[(Z)-5-Hydroxy-3-methylpent-3-enyl]-1,4a-dimethyl-6-methylidene-3,4,5,7,8,8a-hexahydro-2H-naphthalene-1-carboxylic acid	1.77	0.00773	0.5504	down
Citraconic acid	1.59	0.01960	0.5512	down
Acetophenone	1.73	0.00744	0.5552	down
Annosquamosin-B	1.81	0.00309	0.5588	down
PI(16:0/18:1)	1.93	0.00170	0.5594	down
5-Methyl DL-glutamate	1.52	0.03392	0.5619	down
3,4-Methylenedioxy-alpha-pyrrolidinopropiophenone	1.81	0.00685	0.5630	down
Hexamethylquercetagetin	1.41	0.04389	0.5635	down
5-Bromo-6-chloro-1H-indazole	1.62	0.01711	0.5696	down
2-[4-(4-Nitro-2,1,3-benzoxadiazol-5-yl)-1-piperazinyl]ethanol	1.45	0.03490	0.5704	down
2-Linoleoyl-1-palmitoyl-sn-glycero-3-phosphoethanolamine	1.73	0.00871	0.5747	down

Nalpha-Methylhistidine	1.95	0.00135	0.5757	down
Hippuric acid	1.52	0.03504	0.5769	down
Cyclamic acid	1.52	0.03504	0.5769	down
Oxeladin	1.87	0.00371	0.5783	down
Isorhamnetin	1.43	0.04533	0.5790	down
2,4,6-Trihydroxydihydrochalcone	1.79	0.00618	0.5819	down
Epinephrine	1.70	0.01333	0.5866	down
Thr-Leu	1.97	0.00116	0.5877	down
Salbutamol	1.90	0.00224	0.5878	down
8-Hydroxy-7(11)-eremophilen-12,8-olide	1.52	0.03314	0.5886	down
Kanokoside-D	1.77	0.00648	0.5892	down
PE(15:0/18:2(9Z,12Z))	1.46	0.03831	0.5893	down
Leu-Leu	1.69	0.01128	0.5917	down
Ile-Leu	1.69	0.01128	0.5917	down
2-Phenyl-4-(1-pyrrolidinyl)quinazoline	1.59	0.02071	0.5920	down
Cinncassiol-E	1.44	0.03997	0.5949	down
Phaeophorbide-b	1.95	0.00168	0.5963	down
(3-Oxo-2-piperazinyl)acetic acid	1.72	0.01193	0.5975	down
PC(35:1)	1.63	0.01718	0.5987	down
(4OH,8Z,t18:1)-sphingosine	1.68	0.01196	0.5996	down
4-Hydroxyphenylpyruvic acid	1.69	0.01061	0.6017	down
(S)-Nerolidol-3-O-[a-L-rhamnopyranosyl-(1->4)-a-L-rhamnopyranosyl-(1->6)-b-D-glucopyranoside]	1.67	0.01340	0.6020	down
Coumestrol	1.61	0.01810	0.6021	down
Corypalline	1.46	0.03527	0.6069	down
4-Hydroxy-2-oxo-heptanedioate	1.52	0.03318	0.6072	down
3-Caffeoylpelargonidin-5-glucoside	1.99	0.00152	0.6111	down
Ile-Thr	1.76	0.00837	0.6122	down
Leu-Thr	1.55	0.02439	0.6122	down
Dihydrouracil	1.58	0.01850	0.6129	down
3-Pyridinemethanol	1.75	0.00929	0.6147	down
3-Hydroxy-2-methylpyridine	1.75	0.00929	0.6147	down
Ile-Asn	1.73	0.01076	0.6175	down
PE(34:2)	1.72	0.01102	0.6191	down
13(S)-HODE	1.42	0.04719	0.6194	down
N,N-Dimethylarginine (ADMA)	1.94	0.00116	0.6213	down

(2R)-Amino(4-hydroxyphenyl)ethanoic acid	1.63	0.02152	0.6215	down
8,9-Dihydroxy-5Z,11Z,14Z-eicosatrienoic acid	1.56	0.02261	0.6224	down
[1-hydroxy-1-(4-methoxyphenyl)propan-2-yl] 4-methoxybenzoate	1.60	0.01718	0.6247	down
Gln-Ile	1.48	0.03152	0.6285	down
Ethoxyacetic acid	1.66	0.01601	0.6289	down
5-Methylcytosine	1.67	0.01539	0.6322	down
Prostaglandin-PGE2-1-glyceryl-ester	1.45	0.03738	0.6346	down
2-(4,4-Difluorocyclohexyl)ethan-1-amine	1.85	0.00314	0.6354	down
Betaine	1.73	0.01046	0.6376	down
Tyr-Ile	1.47	0.04023	0.6447	down
7-Aminonimetazepam	1.32	0.04874	0.6453	down
Psoralen	1.62	0.01665	0.6499	down
4-Chloro-2,6-bis(hydroxymethyl)phenol	1.61	0.01653	0.6513	down
3-METHYLGLUTACONIC ACID	1.55	0.02878	0.6530	down
5-[1-(Phenylmethyl)-1H-indazol-3-yl]-2-furanmethanol	1.94	0.00224	0.6591	down
Synephrine	1.88	0.00274	0.6601	down
M146T178	1.55	0.02996	0.6607	down
2-Ketobutyric acid	1.68	0.01455	0.6617	down
[1-(5-fluoropentyl)-1H-benzimidazol-2-yl]-1-naphthalenyl-Methanone	1.65	0.01301	0.6625	down
19-Noretiocholan-3b-ol-17-one	1.67	0.01223	0.6634	down
3-Amino-2-oxazolidinone	1.90	0.00278	0.6657	down
(5alpha)-Androst-2-en-17-one	1.67	0.01134	1.5151	up
2-(4-Nitrophenyl)butyric acid	1.69	0.01099	1.5244	up
M488T53	1.35	0.04514	1.5254	up
7,12-Dioxolithocholic acid	1.43	0.03113	1.5314	up
tras-4-Hydroxycinnamic acid sulfate	1.48	0.02749	1.5338	up
4-Methoxy-2,4-dioxobutanoic acid	1.74	0.00669	1.5366	up
Terminolic acid	1.74	0.00616	1.5370	up
2-Acetoxy-3-geranylgeranyl-1,4-dihydroxybenzene	1.81	0.00508	1.5402	up
Mangostine	1.74	0.00632	1.5405	up
Cavipetin-C	1.52	0.02707	1.5527	up
[3-(Trifluoromethyl)-1H-pyrazol-1-yl]acetic acid	1.50	0.02566	1.5601	up
Polyporusterone-D	1.58	0.01640	1.5622	up
4-[5-(7-Hydroxy-5,5,8a-trimethyl-2-methylidene-3,4,4a,6,7,8-hexahydro-1H-naphthalen-1-yl)-3-methylpentoxy]-4-oxobutanoic acid	1.52	0.02268	1.5701	up

Achalensolide	1.39	0.04963	1.5770	up
UDP-6-sulfoquinovose	1.58	0.01693	1.5907	up
Pallidol	1.70	0.00766	1.5913	up
S-(2-Hydroxyethyl)glutathione	1.78	0.00460	1.5914	up
20-Oxo-5-O-beta-mycaminosyltylactone	1.54	0.02894	1.6010	up
3-Hydroxy-5-((3-hydroxy-6-methyl-5-(pentofuranosyloxy)octanoyl)oxy)-6-methyloctanoic acid	1.77	0.00659	1.6022	up
Sempervirine cation	1.67	0.00886	1.6049	up
M384T137	1.35	0.04360	1.6063	up
Flavone base + 4O, 1Prenyl	1.92	0.00178	1.6079	up
beta-Muricholic acid	1.90	0.00155	1.6156	up
Ethyl nitroacetate	1.36	0.03937	1.6186	up
Triamcinolone	2.00	0.00071	1.6224	up
Hexobarbital	1.72	0.00590	1.6225	up
N-Nonanoyl-L-homoserine lactone	1.49	0.02042	1.6260	up
Asarinin	1.92	0.00235	1.6309	up
Parvisoflavone B	1.50	0.03554	1.6321	up
Melamine	1.52	0.02750	1.6347	up
5-[(Z)-16-(3,5-Dihydroxyphenyl)hexadec-12-enyl]benzene-1,3-diol	1.59	0.01393	1.6352	up
(1R,4aR,4bS,6aS,9R,10R,10aS)-1-(Carboxymethyl)-10-hydroxy-2-(1-methoxy-2-methyl-1-oxopropan-2-yl)-1,4a,4b,9,10-pentamethyl-3,4,5,6,7,8,9,10a,12,12a-decahydro-2H-chrysene-6a-carboxylic acid	1.71	0.00675	1.6360	up
M462T74	1.95	0.00091	1.6376	up
9-Methylxanthine	1.51	0.02683	1.6388	up
Gilvocarcin V	1.49	0.03287	1.6392	up
M211T92	1.59	0.01603	1.6488	up
5-Bromo-4-fluoro-2-hydroxybenzoic acid	1.50	0.02913	1.6591	up
Ganolucidic-acid-D	1.68	0.00740	1.6631	up
(2Z)-3-[2-(beta-D-glucopyranosyloxy)-4-methoxyphenyl]-2-Propenoic acid	1.96	0.00100	1.6647	up
o-Nitrobenzoate	1.64	0.01141	1.6672	up
4-[3-Methyl-5-(5,6,7-trihydroxy-1,2,4a,5-tetramethyl-3,4,6,7,8,8a-hexahydro-2H-naphthalen-1-yl)pentoxy]-4-oxobutanoic acid	1.46	0.03101	1.6698	up
14alpha-Hydroxypaxilline	1.66	0.00801	1.6819	up
Clethodim	1.50	0.02595	1.6951	up
Dihydromyricetin	1.76	0.00628	1.6953	up
5-Methoxybilobetin	1.62	0.01191	1.6955	up
M513T62	2.17	0.00002	1.6965	up

Barbital	1.47	0.03198	1.6979	up
4-Bromo-6-fluoro-2-methylquinoline	1.49	0.02327	1.7026	up
Etoposide	1.54	0.01680	1.7053	up
6-Chloro-7-fluoroisatin	1.43	0.03306	1.7055	up
M214T70	1.57	0.02304	1.7060	up
N-Acetylaspartylglutamate	1.41	0.03336	1.7186	up
SM(d18:0/16:0)	1.44	0.04135	1.7277	up
M446T73	1.84	0.00269	1.7362	up
5-Nitro-2-(2-pyridinyl)-1H-benzimidazole	1.61	0.01461	1.7481	up
Carnosol	1.53	0.02249	1.7528	up
M250T113	1.51	0.02461	1.7668	up
3-Oxocholic acid	1.53	0.02793	1.7670	up
7-Ketodeoxycholic acid	1.53	0.02793	1.7670	up
7-Chloro-8-methyl-4-hydroxy-3-quinolinecarboxylic acid	1.39	0.04012	1.7724	up
1-a,24R,25-Trihydroxyvitamin-D2	1.97	0.00052	1.7737	up
N-(4-Methylphenyl)-N-(6-methyl-2-pyridinyl)thiourea	1.61	0.01550	1.8048	up
M766T223	1.99	0.00063	1.8189	up
Butyl-oleate-sulfate	1.76	0.00575	1.8203	up
Ixabepilone--	1.63	0.01451	1.8240	up
8-Methoxy-4-oxo-1,4-dihydroquinoline-2-carboxylic acid	1.50	0.02758	1.8390	up
PD 169316	1.39	0.04364	1.8545	up
N-Methyl-gamma-oxo-3-pyridinebutanamide	1.42	0.03506	1.8666	up
M567T99	1.86	0.00231	1.8730	up
Caffeoylcholine-	1.78	0.00582	1.8847	up
Benzamide	1.67	0.00999	1.8866	up
Isovaleric acid	2.09	0.00027	1.9037	up
Valeric acid	2.09	0.00027	1.9037	up
6-(3-Bromoanilino)-2,4(1H,3H)-pyrimidinedione	1.57	0.01652	1.9226	up
ISOPALMITIC ACID	1.52	0.02699	1.9284	up
2-hydrazinyl-5-nitro-Pyridine	1.62	0.01217	1.9444	up
5-(4-Fluoro-3-nitrophenyl)-1H-tetraazole	1.60	0.01230	1.9641	up
Neolinustatin	1.93	0.00165	1.9690	up
3,4-Dihydroxy-4-(4-methoxyphenyl)-1,3-dihydroquinolin-2-one	1.78	0.00607	1.9768	up
Austalide-J	1.40	0.04475	1.9799	up
Dixyrazine	1.56	0.01923	2.0049	up

Butaprost (free acid)	1.38	0.04546	2.0084	up
N1,N8-Diacetylspermidine	1.65	0.01479	2.0229	up
2-[(6-Amino-9H-purin-8-yl)thio]acetic acid	1.65	0.01157	2.0294	up
Lys-Arg	1.78	0.00659	2.1083	up
Monensin sodium salt	1.71	0.00901	2.1254	up
Homoarecoline	1.62	0.01681	2.1326	up
Ganoderic acid A	1.46	0.03877	2.1591	up
Ganoderic acid B	1.46	0.03877	2.1591	up
D-erythro-Sphingosine C-20	1.88	0.00334	2.1786	up
M263T84	1.66	0.01055	2.1975	up
2-Angeloyl-9-(3-methyl-2E-pentenoyl)-2b,9a-dihydroxy-4Z,10(14)-oplopadien-3-one	1.81	0.00523	2.2077	up
M359T106	1.58	0.01605	2.2554	up
Glucosyl-passiflorate	1.45	0.03197	2.2805	up
2-(3-Chlorophenyl)-4-methyl-1H-imidazole-5-carboxylic acid	1.70	0.00941	2.2908	up
M475T187	1.54	0.02222	2.2970	up
(4R)-4-((3R,5R,6S,7R,9S,10R,12S,13R,14S,17R)-3,6,7,12-tetrahydroxy-10,13-dimethylhexadecahydro-1H-cyclopenta[a]phenanthren-17-yl)pentanoic acid	2.08	0.00019	2.3433	up
M430T184	1.81	0.00359	2.3655	up
Nalidixic Acid	1.63	0.01375	2.3673	up
(R)-4-((1R,3S,5S,7R,8S,9S,10S,12S,13R,14S,17R)-1,3,7,12-tetrahydroxy-10,13-dimethylhexadecahydro-1H-cyclopenta[a]phenanthren-17-yl)pentanoic acid	1.70	0.00903	2.3758	up
Mardepodect	1.64	0.01382	2.3823	up
M648T197	1.71	0.00687	2.3875	up
13,14-Dihydro-15-ketotetranorprostaglandin F1beta	1.73	0.01016	2.4557	up
Suberylglycine	1.67	0.01057	2.4637	up
N-3-Oxohexadec-11Z-enoyl-L-homoserine lactone	1.95	0.00100	2.4874	up
M490T54	1.71	0.00775	2.5157	up
Ethyl 2-(3-chloropyridin-2-yl)-5-oxopyrazolidine-3-carboxylate	1.74	0.00619	2.5231	up
M363T44	1.60	0.01682	2.5238	up
4-(4-Phenyl-5-sulfanyl-4H-1,2,4-triazol-3-yl)phenol	1.36	0.04339	2.5620	up
M554T75	1.48	0.03353	2.6041	up
2,3-Bis-O-(geranylgeranyl)glycerol 1-phosphate	2.04	0.00028	2.6152	up
PC(34:3)	1.42	0.04540	2.6223	up
Heptadecasphinganine	1.62	0.01653	2.6707	up
LPI(17:1)	1.42	0.03938	2.6761	up
Sph(d18:0)	1.64	0.01633	2.7224	up

Indoleacetic acid	1.82	0.00301	2.7406	up
O-Desmethylastemizole	1.58	0.02065	2.7634	up
Sphingosine	1.43	0.04437	2.7719	up
N-Methyl-1-deoxynojirimycin	1.80	0.00479	2.7921	up
4-Amino-N-(2-fluorophenyl)benzenesulfonamide	1.61	0.01459	2.8483	up
Ethynodiol-diacetate	1.67	0.01011	2.8547	up
3,5-Dimethyluracil	1.94	0.00100	2.8931	up
Methyl 3-(acetyloxy)-16,19-dihydroxy-4,4,8,12,16-pentamethyl-15,17-dioxoandrost-11-ene-14-carboxylate	1.55	0.02296	2.9096	up
Methylimidazoleacetic acid	2.03	0.00036	2.9153	up
5-(Chloromethyl)-7-oxo-4,7-dihydropyrazolo[1,5-a]pyrimidine-3-carbonitrile	1.80	0.00447	3.1215	up
Folate	1.40	0.04305	3.2494	up
5-Methyl-2-(4-methylphenyl)-1H-benzimidazole	2.02	0.00062	3.2570	up
Nigericin	2.07	0.00022	3.2809	up
N-[2-(cyclohexylamino)-1-(2-methylphenyl)-2-oxoethyl]-N-(3-fluorophenyl)-2-methyl-1H-Imidazole-1-acetamide	1.78	0.00618	3.5331	up
N-(4-Methoxy-2-nitrophenyl)isonicotinamide	1.79	0.00531	3.5966	up
PA(12:0/0:0)	1.88	0.00249	3.6250	up
Thr-Met	1.56	0.01968	3.6922	up
3,3-Dimethyl-2-oxobutanoic acid	2.13	0.00012	4.1086	up
7-Diethylamino-3-phenylcoumarin	1.63	0.01407	4.3207	up
Pivagabine	1.57	0.01865	4.3324	up
5-Amino-3-pyrrolidin-1-yl-1H-pyrazole-4-carbonitrile	2.02	0.00062	4.4243	up
2-N-Acetyl-6-deamino-6-hydroxyneomycin C	1.86	0.00232	4.5490	up
Atractylenolide III	1.91	0.00135	4.6091	up
Isopongaflavone	1.38	0.04735	4.9140	up
4-Hydroxyphenyl 6-O-(2-methylbutanoyl)-beta-D-glucopyranoside	1.49	0.02855	4.9544	up
4-Hydroxyphenylacetic acid	1.98	0.00106	5.0901	up
3-Hydroxyphenylacetic acid	1.98	0.00106	5.0901	up
M337T37	1.42	0.04441	5.6374	up
N-Stearoylsphinganine	1.60	0.01696	6.6195	up
HQNO	1.77	0.00494	8.2664	up
Propranolol	1.77	0.00494	8.2664	up
M431T170	1.73	0.00721	8.5964	up
Cer(d18:0/16:0)	1.50	0.02966	9.8837	up
Phenylacetic acid	2.32	0.00000	10.4070	up
2-Methylbenzoic acid	2.32	0.00000	10.4070	up

4-Hydroxyphenylacetaldehyde	2.32	0.00000	10.4070	up
4-Hydroxyacetophenone	2.32	0.00000	10.4070	up
3-Hydroxyacetophenone	2.32	0.00000	10.4070	up
4-Methylbenzoic-acid	2.32	0.00000	10.4070	up
Resiniferatoxin	1.44	0.03659	10.5810	up
Oxalicine B	1.47	0.03598	15.0560	up
Win 64821	1.59	0.01827	40.6110	up

VIP, variable importance in projectio; EE, early estrus; DI, diestrus
